# Transcriptomic Response to 1,25-Dihydroxyvitamin D in Human Fibroblasts with or without a Functional Vitamin D Receptor (VDR): Novel Target Genes and Insights into VDR Basal Transcriptional Activity

**DOI:** 10.3390/cells8040318

**Published:** 2019-04-05

**Authors:** Pedro L. F. Costa, Monica M. França, Maria L. Katayama, Eduardo T. Carneiro, Regina M. Martin, Maria A. K. Folgueira, Ana C. Latronico, Bruno Ferraz-de-Souza

**Affiliations:** 1 Laboratorio de Endocrinologia Celular e Molecular LIM-25 e Unidade de Doencas Osteometabolicas, Divisao de Endocrinologia, Hospital das Clinicas HCFMUSP, Faculdade de Medicina, Universidade de Sao Paulo, Sao Paulo, SP 01246-903, Brazil; pedro.luis_12@hotmail.com (P.L.F.C.); monicaufop@yahoo.com.br (M.M.F.); edu.teodoro@hotmail.com (E.T.C.); 2 Laboratorio de Hormonios e Genetica Molecular LIM-42, Divisao de Endocrinologia, Hospital das Clinicas HCFMUSP, Faculdade de Medicina, Universidade de Sao Paulo, Sao Paulo, SP 05403-900, Brazil; reginamm@usp.br (R.M.M.); anaclusp@gmail.com (A.C.L.); 3 Departamento de Radiologia e Oncologia, Instituto do Cancer do Estado de Sao Paulo (ICESP), Faculdade de Medicina FMUSP, Universidade de Sao Paulo, Sao Paulo, SP 01246-000, Brazil; maria.katayama@fm.usp.br (M.L.K.); maria.folgueira@fm.usp.br (M.A.K.F.)

**Keywords:** vitamin D, calcitriol, microarray, gene expression, CYP24A1, cell proliferation

## Abstract

The vitamin D receptor (VDR) mediates vitamin D actions beyond bone health. While VDR activation by 1,25-dihydroxyvitamin D (1,25D) leads to robust transcriptional regulation, less is known about VDR actions in the absence of 1,25D. We analyzed the transcriptomic response to 1,25D in fibroblasts bearing a severe homozygous hereditary vitamin D resistant rickets-related p.Arg30* VDR mutation (MUT) and in control fibroblasts (CO). Roughly 4.5% of the transcriptome was regulated by 1,25D in CO fibroblasts, while MUT cells without a functional VDR were insensitive to 1,25D. Novel VDR target genes identified in human fibroblasts included bone and cartilage factors *CILP*, *EFNB2*, and *GALNT12*. Vehicle-treated CO and MUT fibroblasts had strikingly different transcriptomes, suggesting basal VDR activity. Indeed, oppositional transcriptional effects in basal conditions versus after 1,25D activation were implied for a subset of target genes mostly involved with cell cycle. Cell proliferation assays corroborated this conjectured oppositional basal VDR activity, indicating that precise 1,25D dosage in target tissues might be essential for modulating vitamin D actions in human health.

## 1. Introduction

The vitamin D receptor (VDR, NR1I1) is a nuclear receptor transcription factor that acts as the main physiologic transducer of vitamin D actions at the cellular level [1,2]. Members of the nuclear receptor superfamily are selectively expressed in target tissues, where they characteristically modulate gene expression by direct binding to DNA regulatory elements upon activation by specific steroid ligands [3]. VDR activation by its high-affinity ligand 1,25-dihydroxyvitamin D (1,25D) leads to transcriptional regulation of numerous target genes and modification of the cellular transcriptomic profile [4,5,6]. The concerted endocrine effects of vitamin D have been chiefly studied in regard to calcium homeostasis, where VDR activation by 1,25D in the intestine, for example, results in increased expression of TRPV6 membrane calcium channels, leading to enhanced calcium absorption in response to the vitamin D signal [7,8].

Along with the elucidation of VDR-mediated vitamin D signaling mechanisms in classic vitamin D target organs such as intestine and bone, VDR expression in several cell types beyond those directly involved in mineral homeostasis have prompted great interest [1]. On one hand, VDR expression in such tissues has provided a solid ground for investigating multiple vitamin D actions across human health besides the regulation of bone and mineral homeostasis, particularly in light of well demonstrated extrarenal 1α-hydroxylase activity and consequent 1,25D availability in a paracrine or autocrine fashion [9,10,11]. On the other hand, the unliganded VDR could be acting in these cells in the absence of 1,25D stimulus, regulating basal gene expression or in response to other stimuli. Indeed, based on phenotypic observation of human and mice VDR defects, a role for the unliganded VDR has been identified in, for example, the hair follicle [1,12].

Related NR1 nuclear receptor subfamily members thyroid hormone receptors alpha (THRA, NR1A1) and beta (NR1A2) have important physiologic actions in the absence of their ligand; they basally repress target gene expression, which results in amplified induction of transcription once triiodothyronine is present [13]. Similar mechanisms have not yet been firmly demonstrated for the unliganded human VDR. Understanding VDR actions in the absence of 1,25D can also shed light on the transcriptional outcome of 1,25D dosage variation and, therefore, the molecular consequences of vitamin D insufficiency, a contemporary health concern [14]. While several studies have investigated cell- and/or tissue-specific VDR-mediated response to 1,25D [15,16,17,18], basal VDR actions in the absence of 1,25D have been scarcely studied in humans.

The human VDR is a 427 amino acid protein with a typical nuclear receptor-type structure composed by a DNA-binding domain (DBD, residues 21 to 96) and a ligand-binding domain (LBD, residues 123 to 427) [19]. VDR mutations lead to hereditary vitamin D-resistant rickets (HVDRR), an autosomal recessive disorder characterized by early-onset hypocalcemic rickets typically accompanied by alopecia; in general, mutations in the DBD lead to more pronounced phenotypes [19]. A male patient with HVDRR caused by a severe VDR defect (p.Arg30*) has been followed in our institution for nearly two decades, allowing us to record the long term outcome of this disease [20,21]. Given the nature of his homozygous molecular defect, introducing a stop codon at the beginning of the transcript caused a severely truncated protein lacking both DBD and LBD, thus VDR function was abolished in his cells, providing an opportunity to analyze a natural human cell model lacking the VDR.

Here, we report the comparative transcriptome analysis of control (wild type VDR) and mutant (p.Arg30* VDR) human fibroblasts with or without 1,25D stimulus as a means to expand our understanding of basal VDR actions. A surprisingly oppositional transcriptional activity was implied and supported by biological evidence of distinct cell proliferation behavior.

## 2. Materials and Methods

### 2.1. Reagents and Cell Culture

To begin, 1α,25-dihydroxyvitamin D3 (D1530, Sigma Aldrich, St Louis, MO, USA) was reconstituted in ethanol (10 µM stock solution). Human skin fibroblasts from the HVDRR patient with a homozygous p.Arg30* VDR mutation [21,22] and from an age/sex-matched control were obtained from 4 mm punch biopsies of the forearm skin after institutional board approval and with informed consent. Skin explants were fragmented in 6-well tissue culture plates and covered in complete AmnioMAX^™^ C-100 medium (Gibco for Thermo Fisher, Waltham, MA, USA) until attachment to surface. After approximately 12 days, fibroblasts grown out of explants covered the well surfaces completely. Secondary fibroblast cultures were maintained in high glucose Dulbecco’s Modified Eagle Medium supplemented with 10% fetal bovine serum and 1% penicillin/streptomycin. Fibroblasts were used for experiments between passages five to fifteen. The entire *VDR* coding sequence (Ensembl transcript ENST00000395324) was verified by Sanger sequencing in control (CO) fibroblasts expressing the wild-type VDR and mutant (MUT) fibroblasts expressing the p.Arg30* VDR (Supplementary Figure S1).

### 2.2. Immunocytochemistry

Approximately 10^4^ cells were seeded onto coverslips with appropriate cell culture medium and fixed with 3.7% formaldehyde. Immunostaining was performed using a mouse monoclonal antibody against human vimentin (V9, sc-6260, Santa Cruz Biotech, Dallas, TX, USA) at 1:200 dilution, and the Mouse ExtrAvidin® Peroxidase Staining Kit (Sigma-Aldrich; secondary antibody at 1:300 dilution). Human umbilical vein endothelial cells (HUVEC) and primate kidney fibroblastoid COS-1 cells were used as negative and positive controls for vimentin, respectively.

### 2.3. Microarray Analysis of Global Gene Expression

Global gene expression of CO and MUT fibroblasts in response to treatment with 1,25D or ethanol vehicle (veh) was analyzed using microarrays. Six independent biological replicates were performed for each experimental condition: CO Veh, CO 1,25D, MUT Veh, and MUT 1,25D. Cells were grown in 6-well plates and treated with 10 nM 1,25D or ethanol (1 μL/mL of medium) for 24 h before RNA extraction using the RNeasy Protect Cell Mini Kit (Qiagen, Valencia, CA, USA). Based on quality control of extracted RNA performed with the 2100 Bioanalyzer (Agilent Technologies, Palo Alto, CA, USA), four samples of each condition were chosen for microarray gene expression analysis, all with RNA integrity number above 8.00. Samples were processed using Ambion® WT Expression kit (Thermo Fisher) and GeneChip WT Terminal Labeling (Affymetrix, Santa Clara, CA, USA) kits according to manufacturer’s instructions, starting with 200 ng total RNA. Altogether, sixteen samples of labeled fragmented cDNA were hybridized to GeneChip Human Gene 2.0 ST Arrays (Affymetrix). Array data were analyzed using Partek Genomics Suite® (Partek Inc, St Louis, MO, USA), and based on quality control, one array (MUT Veh) was excluded. A Benjamini-Hochberg-corrected *p*-value cut-off of 0.05 was used for selecting significant differentially expressed genes. Functional annotation enrichment analysis for subsets of target genes was performed using DAVID Bioinformatics Resources v6.7 (https://david.ncifcrf.gov/) [23]. The array data were submitted to the National Center for Biotechnology Information Gene Expression Omnibus (http://www.ncbi.nlm.nih.gov/geo) under series accession no. GSE127314.

### 2.4. Validation by Quantitative RT-PCR

Next, 1,25D-dependent changes in transcript levels of selected target genes in CO and MUT fibroblasts were assessed by quantitative RT-PCR. First-strand cDNA was generated using SuperScript III reverse transcriptase (Thermo Fisher) and quantitative PCR performed in a StepOnePlus Real-Time PCR System (Thermo Fisher) using the following inventoried TaqMan® assays (Thermo Fisher): *CYP24A1* Hs00167999 _m1, *TGFB2* Hs00234244_m1, *RASSF2* Hs00248129_m1, *CILP* Hs00173647_m1, *STC1* Hs00174970_m1, *SPP1* Hs00959010_m1, *GALNT12* Hs00226436_m1, *HBEGF* Hs00181813_m1, *EFNB2* Hs00187950_m1, and *EGR1* Hs00152928_m1. At least three independent experiments in triplicates were performed. Relative quantitation normalized by endogenous control *GAPDH* was performed using the 2^−ΔΔCT^ method with 95% confidence level on StepOne software v2.3 (Thermo Fisher).

### 2.5. Cell Proliferation Assays

Proliferation of CO and MUT fibroblasts in response to 1,25D or vehicle was analyzed by two methods—direct automated cell counting in trypan blue exclusion assays and indirect cell viability assays through colorimetric analysis of media formazan content. Analyses were performed 4, 7, 10, or 14 days after cell seeding, and treatment media containing 1,25D or vehicle were changed on D4, D7, and D10. For the cell counting assay, cells were seeded at 10^4^ cells/well (6-well plates) and counted at fixed time points in a Countess™ automated cell counter (Life Tech) following exposure to 0.2% trypan blue. For the colorimetric analysis of formazan generation, cells were seeded at 10^3^ cells/well (96-well plates), and the CellTiter 96® AQueous One Solution cell proliferation assay (Promega, Madison, WI, USA) was used at fixed time points. Following a 2 h incubation with the reagent, absorbance at 450 nm was read using a GloMax®-Multi+ Detection System (Promega). All experiments were performed at least three times in triplicates. Statistical analysis with one-way ANOVA and post hoc pairwise comparison by Tukey’s procedure was performed using PASW 17.0 (SPSS Inc., Hong Kong, China); significance level was set at 0.05. 

## 3. Results

### 3.1. Transcriptomic Response to 1,25D in Human Fibroblasts

**Figure 1:**
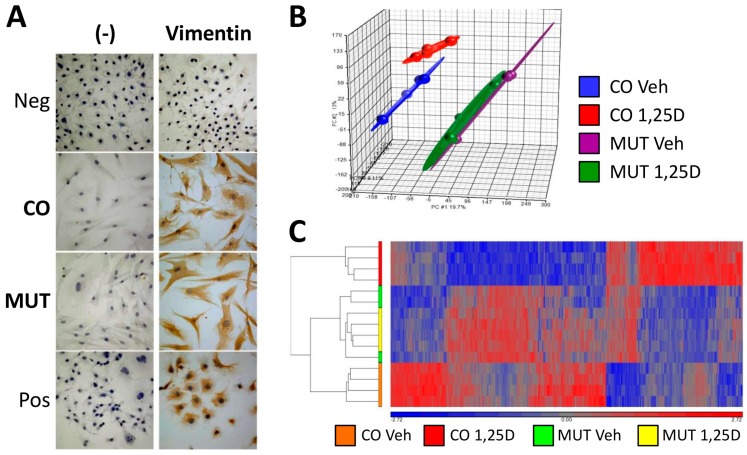
Transcriptomic response to 1,25D in control fibroblasts (CO) and mutant (MUT) fibroblasts. (**A**) Immunocytochemical staining of vimentin was seen in CO and MUT fibroblasts and in COS-1 fibroblastoid cells; CO and MUT cells had the typical fibroblastic morphology with elongated shape and multipolarity (Pos, positive control) but not in human umbilical vein endothelial cells (HUVEC) (Neg, negative control); (-) denotes experiments performed without the primary anti-vimentin antibody for internal control. (**B**) Principal component analysis and (**C**) hierarchical clustering of Human Gene 2.0 ST microarrays corresponding to the four experimental conditions, CO ethanol vehicle (Veh) (n = 4 arrays), CO 1,25D (n = 4), MUT Veh (n = 3) and MUT 1,25D (n = 4), showing markedly different global gene expression patterns between CO fibroblasts treated with vehicle versus 1,25D, and between CO versus MUT fibroblasts in basal state (Veh); As expected, 1,25D did not elicit changes in global gene expression patterns in MUT fibroblasts (MUT 1,25D versus MUT Veh), confirming that vitamin D receptor (VDR) function was abolished in these cells (analyses performed using Partek Genomic Suites).

Secondary in vitro cultures of human fibroblasts expressing wild-type VDR (CO) or the HVDRR-causing p.Arg30* mutant VDR (MUT) were established (Figure 1A). Due to the severe nature of the homozygous *VDR* mutation in MUT fibroblasts, VDR function was expected to be abolished in these cells. Transcriptomic microarray analysis of CO and MUT fibroblasts after 24 h treatment with 1,25D or vehicle showed that treatment with 1,25D had very little effect on global gene expression of MUT fibroblasts, as expected, while a pronounced transcriptomic response to 1,25D was seen in CO fibroblast expressing an intact VDR (Figure 1B,C). Notably, global gene expression patterns of CO and MUT fibroblasts in basal state (treatment with vehicle) were markedly different (Figure 1B,C).

In CO fibroblasts with intact VDR, expression of 1178 target genes was regulated by 1,25D (CO 1,25D versus CO Veh, Benjamini-Hochberg *p* < 0.05), including 453 up-regulated and 725 down-regulated genes (Supplementary Table S1). Remarkably, 1,25D changed expression levels of approximately 4.5% of the genome in these cells. If an arbitrary fold change (FC) threshold of 1.5 was used in order to ascertain perhaps more biologically relevant target genes, 126 targets were up-regulated (FC ≥ 1.5) and 94 targets were down-regulated (FC ≤ −1.5) by 1,25D treatment in CO fibroblasts (total of 220 targets, 19% of identified target genes). 

Identified targets regulated by 1,25D-activated VDR included well-known VDR targets such as *CYP24A1*, *TGFB2*, *RASSF2*, and *HBEGF* [24,25] and novel targets such as *CILP*, *GALNT12*, and *EFNB2*. Top-ranking up- or down-regulated target genes identified in response to 1,25D in CO fibroblasts are detailed in Table 1.

Strikingly, only two transcripts were differentially expressed in MUT fibroblasts after treatment with 1,25D (MUT 1,25D versus MUT Veh, Benjamini-Hochberg *p* < 0.05): *EGR1* transcript levels were up-regulated (+2.7-fold change, *p* = 10^−7^), while transcript levels of the predicted gene AC012360.6 were down-regulated (−1.3-fold change, *p* = 10^−6^); 1,25D-dependent regulation of only one known target gene (EGR1) in MUT fibroblasts confirmed their extreme insensitivity to 1,25D due to the lack of a functional VDR.

For a subset of target genes, results of microarray analysis of differential gene expression were confirmed by quantitative reverse transcriptase PCR (qRT-PCR), as shown in Table 2.

### 3.2. Comparative Analysis of Transcriptome Profiling in CO and MUT Fibroblasts

Prompted by the striking difference in global gene expression patterns of vehicle-treated CO and MUT fibroblasts seen in Figure 1B,C, and taking into account the absence of functional VDR in MUT cells, we analyzed differential gene expression between CO Veh and MUT Veh microarrays, finding 4051 differentially expressed genes (Benjamini-Hochberg *p* < 0.05). In order to try and circumvent the potential transcriptomic consequences of further unknown genetic and epigenetic differences between CO and MUT cells, genes that were also differentially expressed after 1,25D treatment (CO 1,25D versus MUT 1,25D) were excluded, rendering 2164 differentially expressed genes that could reflect basal unliganded VDR regulation without 1,25D stimulus in control fibroblasts. This population of putative basal VDR targets was then compared with the 1178 1,25D-activated VDR targets identified in CO fibroblasts (CO 1,25D versus CO Veh), yielding 268 target genes potentially regulated by VDR either with or without 1,25D stimulus (Supplementary Table S2). Remarkably, opposing regulation was seen in 99.9% of these targets (267/268 genes), the majority of which (194/267 genes, 72.6%) were basally upregulated by the VDR and down-regulated by 1,25D-dependent VDR activation (Table 3).

Aiming to extract a biological functional meaning from these populations of target genes, a gene ontology enrichment analysis was performed using the DAVID tool. As seen in Table 4, 1,25D-activated VDR targets were enriched for steroid signaling as well as skeletal development and metabolism, probably reflecting endocrine actions of 1,25D, while putative unliganded VDR targets and combined targets with opposing regulation were mainly involved in cellular processes, such as DNA repair and replication, potentially reflecting basal VDR activity in cell cycle and proliferation. 

### 3.3. Analyses of Cell Proliferation Behaviour Corroborate Opposing Regulation by the VDR

**Figure 2:**
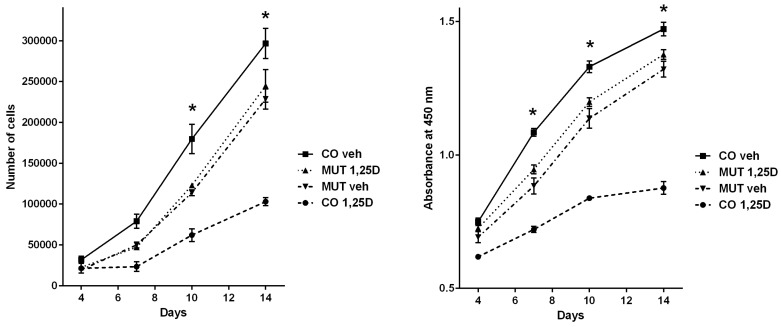
Analyses of proliferation of CO and MUT fibroblasts treated with 1,25D or vehicle. Left panel displays the direct automated cell counting after trypan blue exclusion on D4, D7, D10, and D14. Right panel displays the indirect analyses of cell viability through colorimetric measurement of formazan generation using the CellTiter 96® AQueous One Solution assay on D4, D7, D10, and D14. Treatment of CO fibroblasts with 1,25D significantly decreased cell proliferation (D10 and D14 in both analyses, also on D7 on indirect analysis), while proliferation of MUT fibroblasts was unaffected by 1,25D. Following treatment with vehicle, proliferation of CO fibroblasts was significantly higher than of MUT fibroblasts (D10 and D14 in both analyses, also on D7 on indirect analysis). Vertical bars represent the standard error of the mean (SEM), * one-way ANOVA *p* < 0.05.

Based on functional annotation enrichment analysis evidence of putative basal and combined VDR targets involvement in cell cycle processes, we investigated the proliferation behavior of CO and MUT fibroblasts in basal conditions and after 1,25D activation through direct (trypan blue exclusion automated cell counting) and indirect (colorimetric analysis of formazan generation as a surrogate for cell viability) methods. As shown in Figure 2, proliferation of CO fibroblasts was significantly decreased by 1,25D after 10 days (ANOVA with Tukey’s post hoc test *p* = 4.1 × 10^−7^ and *p* = 1.2 × 10^−11^ for direct and indirect analyses, respectively) and 14 days (*p* = 9.5 × 10^−8^ and *p* = 2.2 × 10^−12^) of 1,25D treatment, while proliferation of MUT fibroblasts was unaffected by 1,25D. However, comparison of CO and MUT fibroblasts treated with vehicle showed that CO fibroblasts proliferated significantly more than MUT fibroblasts in 10 days (*p* = 9.1 × 10^−4^ and *p* = 4.2 × 10^−5^) and 14 days (*p* = 0.023 and *p* = 0.002), suggesting that the unliganded VDR may potentially drive cell proliferation in an opposite direction to 1,25-D-activated VDR.

## 4. Discussion

The identification of the VDR in tissues beyond those directly involved in calcium homeostasis propelled the investigation of extraskeletal actions of vitamin D. Here, we show that in human fibroblasts expressing a functional wild-type VDR, treatment with 1,25D was capable of greatly affecting global transcriptome output, resulting in increased or decreased transcript levels of roughly 4.5% of the genome. Accordingly, 1,25D-activated VDR has been previously shown to regulate the expression of hundreds of genes in different cellular models [1,5,6]. In contrast, we also show that in fibroblasts bearing the HVDRR-related homozygous p.Arg30* VDR mutation (and therefore lacking a functional VDR), global gene expression was virtually unaffected by treatment with 1,25D, proving that most 1,25D-dependent signaling is indeed mediated by the VDR in human fibroblasts. These findings corroborate a notable role for the 1-25D-activated VDR in several human cells and tissues not directly involved with classic endocrine actions of vitamin D in bone and mineral metabolism [2,10].

The identification of *CYP24A1* as the most up-regulated target gene in CO fibroblasts treated with 1,25D supports our experimental design, since this is the best known VDR target in a variety of models [1,26]. We also identified *TGFB2*, *RASSF2*, *SPP1*, and *HBEGF* as relevant 1,25D-activated VDR targets in this system, corroborating previous reports of their regulation by the VDR [24,25,27]. Indeed, comparison of our current results to a previous microarray analysis of 1,25D-treated breast tissue fibroblasts yielded many concordant up-regulated targets [28]. A recent RNA sequence-based transcriptomic analysis of human monocytes explored primary (2.5 h) versus secondary (24 h) 1,25D targets [29]; interestingly, an overlap of relatively unexplored VDR targets *ADAMDEC1*, *IL7R*, and *NPTX1* arose from these datasets in comparison to ours. Notably, several novel targets were identified, warranting investigation of their involvement with 1,25D-regulated processes. Negatively regulated protein glycosylation factor *GALNT12* and bone remodeling-related *EFNB2* and positively regulated cartilage extracellular matrix gene *CILP* seem worthy of future exploration. Finally, with regard to 1,25D-dependent VDR regulation of transcription, a surprising majority of targets (62%) were down-regulated by 1,25D, rather than up-regulated, suggesting a repressive role for 1,25D in human fibroblasts.

While VDR function has been most frequently studied following ligand (1,25D) activation, it may also have important physiologic activity in the absence of vitamin D unliganded. Crucial VDR actions in the hair follicle, for example, have been shown to be independent of 1,25D by several lines of human and rodent evidence [30,31]. More recently, unliganded VDR regulation of *UCP1* and, consequently, adipocyte browning, has been proposed as a mechanism of energy balance modulation [32]. Several other nuclear receptors, most notably the “orphan” members of this transcription factor superfamily, act independently of a specific ligand, paving the way for a deeper exploration of unliganded VDR actions [33].

Interestingly, some nuclear receptors such as the thyroid hormone receptors are capable of opposing regulation of target genes depending on whether their ligand is available or not [34]. Evidence of similar patterns of transcriptional regulation has lately started to emerge with regard to the VDR. Alimirah et al. have reported a repressive action of the unliganded VDR on the expression of target gene *CYP24A1* in breast cancer cells lines in contrast to rapid stimulation of its transcription induced by 1,25D [35]. In 2015, Lee & Wesley Pike reported the analyses of *Vdr* and *Cyp27b1* transgenic mouse models, leading to the observation of lower expression of VDR target genes in presence of unliganded VDR and suggesting a selective suppressor/de-repressor role for the VDR in the absence of 1,25D [36].

The comparative analysis of transcriptome profiling of CO and MUT fibroblasts in basal conditions (i.e., vehicle treatment) provided us an opportunity to look into the contribution of a functional VDR to basal transcriptional output in genomic scale. Indeed, differences in gene expression were remarkable, leading us to identify a population of more than 2000 putative target genes potentially regulated by the unliganded VDR. Once these putative targets were confronted to 1,25D-activated VDR targets, a subgroup of 268 genes regulated by the VDR both in basal conditions and following activation by 1,25D emerged, meaning that approximately a quarter of 1,25D targets in these cells could also be basally regulated by the unliganded VDR.

Strikingly, for 267 of those 268 genes, an opposing direction of regulation was seen—targets that were up-regulated in basal conditions were down-regulated following 1,25D stimulus, and vice versa. As seen in Table 3, even though the magnitude of transcriptional regulation of these combined (basal and 1,25D-dependent) was seemingly low in each scenario, the inverse regulatory direction greatly amplified the effects of ligand signaling. Altogether, these findings corroborate, in a transcriptomic scale, recently identified opposing regulation by the VDR in other settings [35,36] and are supported by well-established similar modus operandi by the thyroid hormone receptors [13].

In order to gather additional evidence supporting this conjectured oppositional regulation by the VDR, we set to investigate the proliferation of CO and MUT fibroblasts in response to 1,25D. Several in vitro and in vivo models have shown anti-proliferative actions of vitamin D [1,2,37], which we too identified in CO fibroblasts expressing wild-type VDR. MUT fibroblasts proliferation was unaffected by 1,25D, proving that this anti-proliferative effect of 1,25D was mediated by the VDR. When we compared proliferation patterns for CO and MUT fibroblasts treated with vehicle, it became apparent that CO fibroblasts proliferated more rapidly than MUT fibroblasts in basal conditions. The slower proliferation rate in the absence of a functional VDR suggests that the unliganded VDR could be basally increasing cell proliferation in control fibroblasts, which would then markedly decrease once 1,25D activates the receptor. Whilst it is tempting to attribute this difference in proliferation patterns solely to the lack of a functional VDR in MUT cells, we cannot be certain whether other factors may be contributing to this observation. Indeed, a major limitation of our analysis is that a potential bias may have been introduced by the different genetic and epigenetic backgrounds of CO and MUT fibroblasts.

In conclusion, we report that 1,25D-activated VDR is capable of regulating roughly 4.5% of the transcriptome of human fibroblasts, and that a broad transcriptional output may also be regulated by the unliganded VDR. Furthermore, an opposing transcriptional activity of the VDR in basal versus 1,25D-activated states emerged mainly in the form of basal up-regulation and ligand-induced down-regulation of target genes. These findings expand our understanding of VDR activity and suggest that, in light of opposing regulation, precise 1,25D dosage in target tissues might be essential for modulating vitamin D actions in human health.

## Figures and Tables

**Table 1:** Top-ranking up- and down- regulated 1,25D-activated VDR target genes in human fibroblasts (CO).

Gene Symbol	Transcript Cluster ID ^1^	Fold Change	Adjusted *p*-Value ^2^
**Up-regulated by 1,25D**			
*CYP24A1*	16920497	87.7	4.8 × 10^−16^
*TGFB2*	16677556	5.6	4.4 × 10^−9^
*RASSF2*	16916901	4.0	1.2 × 10^−10^
*CILP*	16810713	3.9	9.1 × 10^−12^
*OSR2*	17071298	3.9	1.2 × 10^−9^
*STC1*	17075553	3.8	1.4 × 10^−9^
*RGNEF*	16986138	3.5	5.3 × 10^−12^
*NID2*	16792954	3.3	2.8 × 10^−9^
*EFTUD1*	16812475	3.3	1.5 × 10^−9^
*MREG*	16908138	3.1	8.9 × 10^−9^
**Down-regulated by 1,25D**			
*KRTAP1-1*	16844572	−1.9	5.3 × 10^−6^
*LCE1F*	16671065	−1.9	1.1 × 10^−4^
*PLK2*	16996433	−1.9	5.3 × 10^−6^
*LGR5*	16754134	−1.9	1.2 × 10^−4^
*EFNB2*	16780859	−1.9	1.0 × 10^−3^
*TRNAI2*	17005782	−2.2	3.8 × 10^−5^
*B3GALT2*	16697471	−2.2	3.5 × 10^−4^
*C11orf87*	16730967	−2.3	1.0 × 10^−8^
*LOC730755*	16844585	−2.3	9.8 × 10^−11^
*GALNT12*	17087413	−2.7	1.3 × 10^−8^

^1^ Transcript cluster identification by Affymetrix Human Gene 2.0 ST array design, ^2^ Benjamini-Hochberg-corrected *p*-value.

**Table 2:** Quantitative RT-PCR validation of differential gene expression findings.

Target Gene	Differential Expression	Microarray		qRT-PCR TaqMan	
		FC	*p*-Value	RQ	RQ Min-Max
*CYP24A1*	CO 1,25D versus Veh up-reg	87.7	10^−16^	1032.0	852.7–1249.2
*TGFB2*	CO 1,25D versus Veh up-reg	5.6	10^−9^	11.8	9.6–14.5
*RASSF2*	CO 1,25D versus Veh up-reg	4.0	10^−10^	9.0	6.2–13.0
*CILP*	CO 1,25D versus Veh up-reg	3.9	10^−12^	247.0	201.8–302.3
*STC1*	CO 1,25D versus Veh up-reg	3.8	10^−9^	8.2	6.6–10.1
*HBEGF*	CO 1,25D versus Veh down-reg	−1.7	10^−5^	0.44	0.33–0.58
*EFNB2*	CO 1,25D versus Veh down-reg	−1.9	10^−3^	0.61	0.43–0.85
*GALNT12*	CO 1,25D versus Veh down-reg	−2.7	10^−8^	0.36	0.24–0.53
*EGR1*	MUT 1,25D versus Veh up-reg	2.7	10^−7^	3.9	3.6–4.4

FC, fold change; RQ, relative quantitation on StepOne software v2.3, based on the 2^−ΔΔCT^ method; RQ min-max, RQ boundaries at 95% confidence value.

**Table 3:** Selected target genes potentially regulated by the VDR in opposing directions in basal conditions versus following activation by 1,25D.

Target Gene	1,25D-Activated VDR Regulation (CO 1,25D vs. CO veh)		Putative Unliganded VDR Regulation (CO Veh vs. MUT Veh)	
	Fold Change	Adj *p*-Value	Fold Change	Adj *p*-Value
*SERPINB7*	+2.7	3.1 × 10^−7^	−1.9	4.7 × 10^−5^
*CCR1*	+2.1	3.6 × 10^−6^	−1.5	1.6 × 10^−3^
*SPP1*	+2.0	3.7 × 10^−7^	−1.7	7.7 × 10^−6^
*MIR548V*	+1.9	1.2 × 10^−4^	−1.7	9.9 × 10^−4^
*SULF2*	+1.8	2.7 × 10^−7^	−1.8	3.1 × 10^−7^
*BMP6*	+1.7	3.0 × 10^−5^	−2.4	7.7 × 10^−7^
*DNER*	+1.7	7.4 × 10^−7^	−1.4	6.0 × 10^−5^
*APBB1IP*	+1.7	2.7 × 10^−10^	−1.7	6.7 × 10^−10^
*SGIP1*	+1.5	1.4 × 10^−4^	−1.4	1.7 × 10^−3^
*ANGPTL4*	+1.5	6.6 × 10^−7^	−1.7	1.2 × 10^−7^
*DKK2*	−1.5	6.5 × 10^−4^	+1.5	2.5 × 10^−3^
*HOXB5*	−1.5	7.3 × 10^−5^	+1.7	1.9 × 10^−5^
*PITPNM3*	−1.6	1.9 × 10^−5^	+1.5	2.9 × 10^−4^
*PDGFC*	−1.6	1.9 × 10^−8^	+1.9	1.6 × 10^−9^
*SPTLC3*	−1.7	3.3 × 10^−7^	+1.7	1.1 × 10^−6^
*SNORA76*	−1.7	2.4 × 10^−4^	+2.1	2.7 × 10^−5^
*CH25H*	−1.8	1.3 × 10^−4^	+2.0	3.6 × 10^−5^
*MIR4659A*	−1.8	4.1 × 10^−4^	+1.7	8.4 × 10^−4^
*LGR5*	−1.9	1.2 × 10^−4^	+2.9	3.4 × 10^−6^
*EFNB2*	−1.9	1.0 × 10^−3^	+1.9	1.8 × 10^−3^

Adj *p*-value, Benjamini-Hochberg-corrected *p*-value.

**Table 4:** Functional annotation of target gene populations using DAVID Bioinformatics Resources.

Target Gene Population	Direction of Regulation	Gene Ontology Enrichment (n of Targets Involved in Process)	DAVID *p*-Value
1,25D-activated VDR targets	Up-reg	skeletal system development (n = 21)	10^−5^
	Up-reg	steroid metabolic process (n = 16)	10^−5^
	Down-reg	cell cycle (n = 65)	10^−8^
	Down-reg	ribonucleotide binding (n = 110)	10^−6^
Putative basal unligandedVDR targets	Up-reg	DNA repair (n = 77)	10^−23^
	Up-reg	DNA replication (n = 62)	10^−23^
	Up-reg	mRNA processing (n = 65)	10^−13^
	Down-reg	endocytosis & membrane invagination (n = 19)	10^−5^
	Down-reg	cell adhesion (n = 37)	10^−4^
Combined basal and 1,25D-dependent VDR targets	Opposing regulation	cell cycle (n = 34)	10^−8^
		DNA repair and replication (n = 17)	10^−7^
		nucleotide binding (n = 52)	10^−5^

## Supplementary Materials

The following are available online at https://www.mdpi.com/2073-4409/8/4/318/s1. Figure S1, Direct DNA sequencing of VDR exon 3 confirming CO and MUT status of fibroblatsts; Table S1, Full set of up- and down-regulated 1,25D target genes in control fibroblasts meeting adjusted *p*-value <0.05; Table S2, Full set of target genes potentially regulated by the VDR both in basal conditions and after 1,25D stimulus, meeting adjusted *p*-value <0.05.
