# Supplementary material for: Transcriptomic Response to 1,25-Dihydroxyvitamin D in Human Fibroblasts with or without a Functional Vitamin D Receptor (VDR): Novel Target Genes and Insights into VDR Basal Transcriptional Activity
Source: Cells. 2019 Apr 5;8(4):318. doi: 10.3390/cells8040318 (PMC6523947; doi:10.3390/cells8040318)
Supplement: Supplementary file 1 [file cells-08-00318-s001.zip › cells-453826-supplementary-revised-/Cells-453826 MS Supplementary Figure 1.pdf]

cells

MDPI

Transcriptomic response to 1,25-dihydroxyvitamin D in human fibroblasts with or without a functional vitamin D receptor (VDR): Novel target genes and insights into VDR basal transcriptional activity

Pedro L. F. Costa, Monica M. França, Maria L. Katayama, Eduardo T. Carneiro, Regina M. Martin, Maria A. K. Folgueira, Ana C. Latronico, Bruno Ferraz-de-Souza

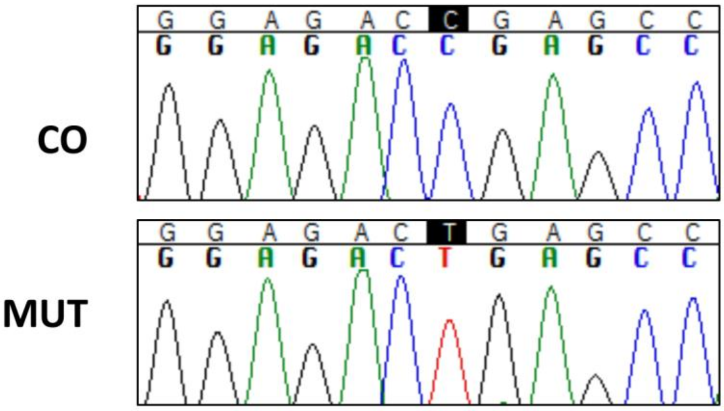

Supplementary Figure 1. Direct DNA sequencing of VDR exon 3 (Ensembl transcript ENST00000395324) confirmed that while control (CO) fibroblasts have the wild-type VDR sequence, mutant (MUT) fibroblasts bear the homozygous c.88C>T variant leading to p.Arg30* VDR.
